# Pneumorrhachis and pneumothorax after epidural analgesia: A case report and review

**DOI:** 10.5339/qmj.2021.1

**Published:** 2021-02-18

**Authors:** Nissar Shaikh, Shoaib Nawaz, Ranjan Mathias, Rahman MA, Marcus Lance, Firdous Ummunissa, Amna Khalifa Tellisi

**Affiliations:** ^1^Department of Anesthesia and Perioperative Care, Hamad Medical Corporation, Doha, Qatar E-mail: snawaz1@hamad.qa; ^2^Department of Obstetrics and Gynecology, Women's Wellness and Research Center, Hamad Medical Corporation, Doha, Qatar; ^3^Department of Obstetrics and Gynecology, Al Wakra Hospital, Hamad Medical Corporation, Al Wakra, Qatar

**Keywords:** epidural analgesia, anesthesia, pneumorrhachis, pneumomediastinum, pneumothorax, respiratory distress

## Abstract

Epidural analgesia or anesthesia is a common procedure for pain relief, especially in obstetrics. Pneumorrhachis and pneumothorax are rare complications of epidural analgesia. They are considered asymptomatic entities but have recently caused increased morbidity and mortality. As the use of epidural analgesia and anesthesia increased significantly in the last decade, clinicians must be aware of this entity. This is a case report of pneumorrhachis causing pneumothorax and pneumomediastinum leading to respiratory distress.

Case: A 26-year-old obese primigravida at 37 weeks’ gestation and with failure of progression of labor underwent lower segment cesarean section under epidural anesthesia. The procedure including the delivery of fetus was uneventful. In the post-anesthesia care unit, the patient became tachypneic, and her oxygen saturation was low despite supplemented oxygen by face mask and adequate analgesia. She was afebrile and was admitted to the surgical intensive care unit (SICU) for further management. In the SICU, incentive spirometry was initiated, and analgesia with intravenous fentanyl was given. Her echocardiogram was normal. Computer tomographic examination ruled out pulmonary embolism but showed pneumorrhachis with extension into the mediastinum and right apical pneumothorax. She was hemodynamically stable. In the next two days, her tachypnea settled, and the oxygen saturation improved to normal. On the third day, she was transferred to the ward and discharged home from there. She was followed up in the outpatient clinic after one and four weeks and was doing well, and her repeat imaging studies were normal.

Conclusion: Epidural analgesia can lead to pneumorrhachis and can cause pneumothorax leading to respiratory distress.

## Introduction

The number of lower segment cesarean section (LSCS) deliveries increased by 18 to 25% in both developed and developing countries with an increasing trend of performing LSCS under regional anesthesia due to obvious advantages from a safety viewpoint.^1^ The commonly used regional anesthesia techniques for LSCS range from spinal to combined spinal epidural and epidural. The frequent complications of these techniques are post-dural puncture headaches, epidural hematoma, epidural abscess, and cardiovascular complications ranging from bradycardia to hypotension and cardiac arrest.^2^ Pneumorrhachis is a rare complication of neuraxial anesthesia, with occasional asymptomatic cases being reported.^3^ Recently, two cases of pneumorrhachis and pneumocephalus leading to cardiac arrest after epidural analgesia were reported by Shin et al.,^4^ but the cause of cardiac arrest was not completely clear. Pneumorrhachis is defined as air within the intradural or extradural compartment of the spinal canal.^5^

To the best of our knowledge, there are no reported cases of pneumorrhachis causing respiratory distress.

This study aimed to report a case of epidural pneumorrhachis causing respiratory distress due to pneumothorax and pneumomediastinum.

Case: A 26-old-year primigravida at 37 weeks’ gestation was admitted to the women's hospital with preeclamptic toxemia. Labetalol infusion was initiated to control her blood pressure. Her comorbidities included hypothyroidism and obesity with a body mass index of 56 kg/m^2^. The induction of labor failed, and the patient was found to have oligohydramnios. It was decided to perform lower section cesarean section (LSCS) to deliver the baby. She was premedicated with H2 blocker, metoclopramide, and sodium citrate. LSCS was performed under epidural anesthesia. The lumbar epidural space was accessed aseptically with the patient in a sitting position, and a single shot of 0.25% bupivacaine (10 mL) was injected after confirming that the position of the tip is in the correct space. The procedure was technically difficult, and the epidural space was accessed in the second attempt. Dorsal 9 to 10 segmental analgesia was achieved, and the surgery was uneventful with minimal blood loss. During the 36-minute procedure, the patient remained stable. A female fetus weighing 2.7 kg was delivered, and the Apgar scores were 9 and 10 at 1 and 5 minutes, respectively. In the postanesthesia care unit, the patient was fully awake, obeyed commands and was hemodynamically stable. Despite receiving a bolus of intravenous fentanyl analgesia, reporting a numerical pain score of one, and maintaining a normal body temperature, the patient was tachypneic (respiratory rate of 36–40/minute). On chest examination, air entry was equal without any added sounds. Immediate chest X-ray did not reveal any abnormalities, and echocardiogram showed normal ejection fraction and no wall motion abnormalities. Her arterial blood gas showed acute respiratory alkalosis (PH 7.49, Pco_2_ 29 mmHg, paO_2_ 110 mmHg, and bicarbonate 23 mmol/L). There had been no attempt of central line insertion.

**Figure 1.**
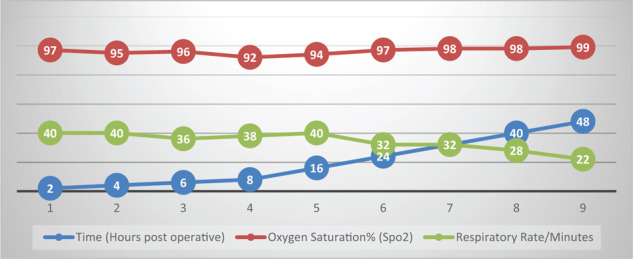
Oxygen saturation and respiratory rate versus timing

The patient was transferred to the surgical intensive care unit for further management. She remained hemodynamically stable, maintaining oxygen saturation of around 97% with 6 L/minute of oxygen supplementation. She was afebrile but persistently tachypneic, despite adequate pain control using patient-controlled analgesia with fentanyl (Figure 1). She received 1 L of intravenous crystalloid (lactated Ringer's solution) during and after the epidural procedure.

**Figure 2.**
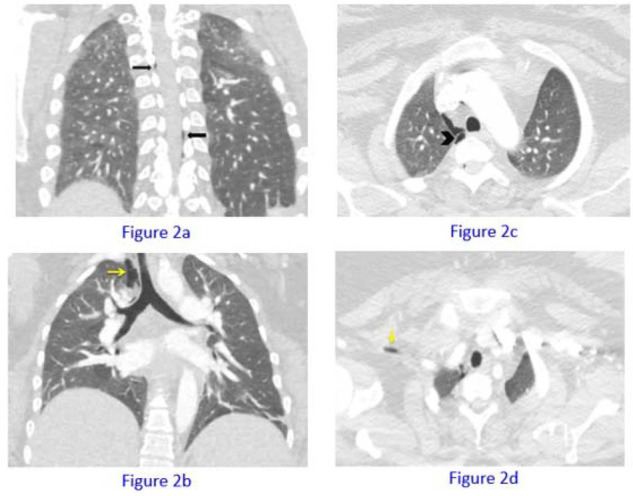
There are subtle linear lucencies (air) noted in the epidural space (black arrows) denoting pneumorrhachis, which developed after thoracic epidural injection. Larger linear lucency along the prevertebral space (arrowhead), with air extending into the right paratracheal region and to the soft tissues of the right base of neck (yellow arrows, particularly along the right subclavian vessels). Overall findings are consistent with iatrogenic pneumomediastinum.

Computerized tomographic (CT) pulmonary angiography ruled out pulmonary embolism, but the CT chest scan showed right apical pneumothorax and pneumomediastinum (Figure 2b–d, arrowhead and black arrow). Air was also detected in the thoracic epidural space causing pneumorrhachis (Figure 2a).

The patient was managed by supportive care, incentive spirometry, and adequate analgesia and was started on an oral diet. She gradually improved, and by the second day, she became stable and was not in respiratory distress anymore. She was transferred to a surgical ward and discharged home from there with instructions to report to the emergency department in case of headache, blurring of vision, epigastric pain, or any signs of wound infections. The patient was followed up in the obstetrics and gynecology outpatient clinic, and she remained stable without any major respiratory or cardiovascular distress, and further imaging studies were normal.

## Discussion

Pneumorrhachis is defined as air within the intradural or extradural compartment of the spinal canal.^5^ It is diagnosed by CT imaging studies. Epidural pneumorrhachis is usually asymptomatic, but the air in the spinal canal can lead to tension pneumocephalus, a life-threatening emergency.^6^ The etiology of epidural pneumorrhachis is iatrogenic, spontaneous, or traumatic. Iatrogenic insertion of air may occur at the time of the loss of resistance (LOR) technique during epidural analgesia.^6,7^ The epidural space is not covered by fascial planes and is in communication with the intervertebral foramina. Air can also enter between the retroperitoneum and epidural space. The driving force of pneumothorax may also push air in the epidural space.^6,7^ Goh^6^ reviewed 13 cases of traumatic pneumorrhachis reported in the literature, and eight cases were secondary to pneumothorax. Iatrogenic pneumorrhachis due to epidural analgesia and anesthesia is considered frequent and asymptomatic,^8,9^ but no case report or case series has been published in the medical literature. This is the first case report of epidural pneumorrhachis causing respiratory distress. As mentioned earlier, the most frequent reported etiology of epidural pneumorrhachis is secondary to trauma, and in this patient, it was mostly due to the use of air for the LOR technique (Loss of resistance) to detect the epidural space. The exact etiopathology of pneumorrhachis extending to the posterior mediastinum and causing pneumothorax is not understood, but the air freely moves through the spinal foramen as there is no fascial barrier; accordingly, the air gets collected in the posterior epidural space because of lesser resistance compared with that in the anterior epidural space (which has a rich vascular network).^9^ This air moves with negative pressure causing pneumomediastinum and possibly pneumothorax. Pneumorrhachis has to be differentiated from free intra-spinal gas collections due to degenerative, malignant, inflammatory, and infectious diseases by gas-forming organisms. The clinical manifestations will guide the differentiation.^8^

Pneumorrhachis usually is asymptomatic, does not tend to migrate, and gets reabsorbed spontaneously and completely. Pneumorrhachis can become symptomatic and associated with discomfort, pain, and even neurological deficits. Cases of traumatic pneumorrhachis leading to sensory symptoms have been reported. Another case report was of a patient with progressive motor deficit of the lower limbs because of entrapped intra-spinal air, which compressed the spinal cord because of the presumptive introduction of air into the intra-spinal arachnoid space after repeated lumbar puncture,^10^ and more recently, there were two more case reports with cardiac arrest following epidural analgesia. This is the first case report of respiratory distress caused by pneumorrhachis.

Since pneumorrhachis is a rare complication with different pathogenesis and etiologies, there are no empiric guidelines for the treatment and standards of care. Pneumorrhachis is considered to be associated with increased morbidity and mortality^4,8,10^; therefore, all the conditions causing pneumorrhachis must be evaluated, and the contributing causes leading to pneumorrhachis have to be appropriately treated. Pneumorrhachis associated with decreased intra-spinal pressure secondary to cerebrospinal fluid leakage usually has a more benign character, whereas entrapped intra-spinal air under pressure entering the cranio-spinal compartment usually in combination with a one-way air valve mechanism might cause tension pneumorrhachis and pneumocephalus with nervous tissue compression requiring intervention. These patients will need interventional therapeutic approach. Our patient responded to conservative therapy. In cases of epidural analgesia and anesthesia, pneumorrhachis can be prevented using saline instead of air for the location of epidural space by the LOR technique.

## Conclusion

This case report concludes that epidural anesthesia may cause pneumorrhachis and pneumothorax and can lead to respiratory distress.

